# Social support and HIV management among people who inject drugs: in-depth interviews in Delhi, India

**DOI:** 10.1186/s12954-025-01185-0

**Published:** 2025-03-14

**Authors:** Rose P. Kaptchuk, Aastha Kant, Surendra S. Shekhawat, Jiban Baishya, Archit Sinha, Ashwini Kedar, Saisha Khanna, Allison M. McFall, Sunil S. Solomon, Shruti H. Mehta, Gregory M. Lucas

**Affiliations:** 1 Johns Hopkins Bloomberg School of Public Health, 615 N Wolfe St, Baltimore, MD 21205 USA; 2 Johns Hopkins University School of Medicine, Baltimore, MD USA; 3 YR Gaitonde Centre for AIDS Research and Education (YRG CARE), Chennai, India; 4 Johns Hopkins Krieger School of Arts and Sciences, Baltimore, MD USA

**Keywords:** Social support, PWID, India, HIV, Harm reduction

## Abstract

**Background:**

People who inject drugs (PWID) experience a higher burden of HIV compared to general populations. Social support has been shown to improve disease management and combat stigma for PWID yet remains unexplored among PWID in low- and middle-income countries.

**Methods:**

We conducted qualitative in-depth interviews to understand social ties and health management among PWID living with HIV in Delhi, India. The research was nested in a factorial randomized controlled trial comparing same-day treatment and community-based care with standard-of-care. Interviews were conducted in Hindi in a private room, audio recorded, transcribed in English, and analyzed inductively using Dedoose.

**Results:**

We conducted 22 interviews (30 min-two hours) with PWID living with HIV in Delhi (all men, ages 21–38 years). 10 slept in houses, 11 on public streets, and one in a shelter. Participants often experienced isolation in their lives but identified avenues of positive social support from healthcare staff, families, peers (friends or injecting partners), and authority figures/public contacts. Healthcare staff provided information and respectful encouragement to manage health. Outreach workers provided support to remind and accompany participants to clinic visits. Family members offered financial support, medicine reminders, and trust. Authority figures/public contacts included employers, shopkeepers, and vendors who provided a safe place to sleep or store belongings, which proved crucial to consistently store and take pills. In some cases, specific social connections created barriers to health by enabling injecting drug use and carrying out harmful behaviors such as physical attacks, disrespect, and theft.

**Conclusion:**

Social connections can offer PWID positive emotional and logistical support to access health services and help them persevere through societal and structural stigmas. However, in some cases they may also contribute negatively to health management challenges. As a harm reduction strategy, public health services can work with PWID to consider untapped opportunities to build positive support and resilience through social ties, as well as how to contend with social connections harmful to health management.

## Background

Globally, the burden of infectious diseases, especially HIV, is higher among people who inject drugs (PWID) compared to general populations. This burden contributes a significant proportion of new HIV diagnoses globally [1]. While there is an estimated 0.6% HIV prevalence among adults, HIV prevalence estimates among PWID globally range from 12.5 to 17.8% [2–4]. A 2023 systematic review estimated a global pooled HIV incidence among PWID of 1.7 per 100 person years with substantial differences by region [5]. In the Southeast Asia region, HIV incidence among PWID was estimated at 3.6 per 100 person years [5]. PWID also often face heightened structural barriers including stigma surrounding drug use, and among those living with HIV, added stigma surrounding HIV [6, 7].

Previous studies have shown that social support can play an important role in managing health and overcoming structural barriers for people with HIV (PWH). Definitions of social support vary but generally refer to perceptions of or experiences of receiving resources from people within one’s social network, including psychological or emotional support as well as material or informational support [8–11]. Although social support is often lacking among PWH [12, 13], studies have highlighted its importance to promote health management among these communities [14, 15]. Social support has been shown to lower depressive symptoms [16, 17], improve physical and cognitive functioning [18], and promote a higher quality of life among PWH [19, 20]. Social support has also been shown to protect against enacted and internalized stigma among PWH [10], yet HIV stigma can make experiencing benefits of social support harder [10]. A 2022 survey among PWH in India demonstrates how higher HIV stigma levels can result in lower social support [21]. Facilitating adherence to medicine that treats infectious diseases can be another important benefit of social support. For example, a 2013 study in sub-Saharan Africa found that social relationships can help maintain antiretroviral therapy (ART) adherence by providing transportation, encouragement, reminders, and social expectations that motivate adherence for PWH [22].

The role of social support among PWID has been less studied, especially in low- and middle-income countries. Yet, existing work among PWID demonstrates that relationships with others can influence housing stability [23], reduce internalized stigma [24], facilitate mental health [25], and improve retention in care [26, 27]. Having people to rely on for social support can also reduce levels of drug use and reduce harms, including overdose [28, 29]. A 2011 study with PWID in India found that living with HIV was negatively associated with receiving emotional support from network members, highlighting how social network factors and HIV stigma can be bidirectional [30]. Pervasive stigma surrounding HIV in India has been identified as a significant predictor of social isolation and depression [21], placing high barriers for PWID living with HIV to benefit from social support.

To better understand these dynamics, we conducted in-depth interviews with PWID living with HIV in Delhi, India. This study aimed to qualitatively explore the role that social connections play in HIV management and how relationships promote or deter wellbeing among PWID.

## Methods

### Study setting

The research was nested in a randomized trial led by Johns Hopkins University in partnership with YRG CARE, an Indian nonprofit working in HIV research and programming. The parent study is a factorial randomized trial to evaluate the impact of same-day ART initiation (vs. standard ART initiation) and community-based HIV treatment (vs. government clinic HIV care) on HIV viral suppression among PWID living with HIV in India (ClinicalTrials.gov Identifier: NCT05165810). Recruitment for the trial in Delhi began in March 2023 and is ongoing as of August 2024. Eligibility criteria for participation in the trial is: being 18 years or older, reporting injection drug use in prior 24 months, and living with HIV with an HIV viral load of 1000 c/mL or higher. To be eligible for an interview, participants had to be enrolled in the trial in Delhi and had to have completed at least one semi-annual study follow-up visit at the time of interview recruitment. All trial participants were cisgender men at the time of interview recruitment, which reflects the demographics of PWID in Delhi and across India [31, 32]. The interviews used for this analysis were designed with a broader scope beyond the topic of this study. They were designed to explore clients’ experiences in the trial and individual-level factors influencing health behaviors, as well as social connections and avenues of support from other people.

### Guiding theories

We consider social support as one component of many levels of factors influencing health and wellbeing of PWID. As outlined in the social ecological model, factors across the individual, interpersonal, organizational, community, and public policy all can play a role in determining health outcomes [33]. A modified social ecological model to guide multiple levels of HIV vulnerability developed in 2013 recognizes that individual level factors (i.e., biologic or behavioral characteristics associated with vulnerability) are insufficient to consider broader community-level HIV dynamics [34]. There are factors outside of an individual’s control which readily influence vulnerability in a given context, such as availability and accessibility of health services, policies, and HIV epidemic stage. In India, past work has documented entrenched systemic barriers to health service access among PWID including long wait times at health centers, judgmental attitudes of healthcare staff, as well as structural barriers including stigma towards PWID and lack of transportation access [35]. While interpersonal networks and social support show potential to help manage these structural challenges, we recognize that social support alone is unlikely to fully determine success in managing HIV and health behaviors among PWID. This social ecologic perspective influenced multiple phases of the study, as we asked about multi-level influences in interview questions and discussed them when interpreting data to consider social support as one factor in a complex environment impacting health and wellbeing.

In considering different types of social support discussed among participants in interviews, we conceptualised support across domains discussed in the social support literature, specifically interpersonal connection (i.e., emotional support and appraisal), informational support, and instrumental support (i.e., access to commodities and resources) [15]. We also engage with the concept of symbiotic support. Social support literature discusses a symbiotic process as interactions which are not just focused on one outcome or benefit, but have multiple outcomes [36]. Some studies consider a symbiotic process as having multiple outcomes for the same individual [37–39], and others in which both parties interacting can benefit [36, 40]. In this study, we consider symbiotic support as a mutual, potentially  beneficial interaction for both people involved. We explored these types of support relating to both individual health management behaviours and access to health services.

### Data collection

We conducted in-depth interviews with clients of the parent trial in Delhi, India in July–August 2023. Interviews were conducted in Hindi by an ethnographer (SSS) based in India uninvolved with the trial implementation. Clients were purposively recruited for a balance across age. Interview guides included questions about avenues of social connections which exist among PWID in Delhi and in what ways these social connections impact health management and wellbeing. Questions surrounding health management focused on behaviors related to the management of HIV, including getting tested, regularly attending clinic visits, treatment adherence, and retrieving treatment refills. Interviews took place in a private room at the trial study site and were audio recorded. All participants provided informed oral consent prior to the interview.

### Analysis

During data collection, the interviewer and the qualitative assessment lead (RPK) had daily de-brief conversations in-person to discuss each interview. They discussed themes and new areas which clients mentioned in interviews and collaborated on what to probe about in subsequent interviews. The qualitative assessment lead wrote memos summarizing their conversations. The interviewer and the qualitative assessment lead engaged in iterative conversations about findings and major themes from the interviews following data collection. The audio recordings of the interviews were transcribed and translated to English. The qualitative assessment lead co-analyzed the data remotely with a qualitative expert (AK) based in India. They read through interviews and generated an inductive codebook based on the topics and perceptions discussed. They coded the interview data using this codebook, engaging in regular discussions about themes and notable differences in preferences and experiences shared by clients across topics. Through discussion of coded data about social connections, they organized support from social networks across three domains discussed in existing social support literature: interpersonal connection, informational support, and instrumental support [15]. They used Dedoose to collaboratively code interviews.

The study was approved by the Johns Hopkins University School of Medicine Institutional Review Board and the YRG CARE Institutional Review Board.

## Results

We conducted 22 interviews lasting between 30 min and two hours. All participants were cisgender men living in Delhi. Ages ranged from 21 to 38 years old (median = 29 years). 10 regularly slept in houses, 11 slept on a public street, and one regularly slept in a shelter. Education ranged from none (n = 5), kindergarten-fifth (n = 7), sixth-eighth (n = 7), to above eighth grade (n = 3). Occupations among respondents included: unemployed; being a driver; working in a wine shop; and various types of daily wage work including garbage collecting, catering, construction, and packing work. Among participants, 19 had initiated ART at the time of the interview.

### Avenues of social connections

**Fig. 1:**
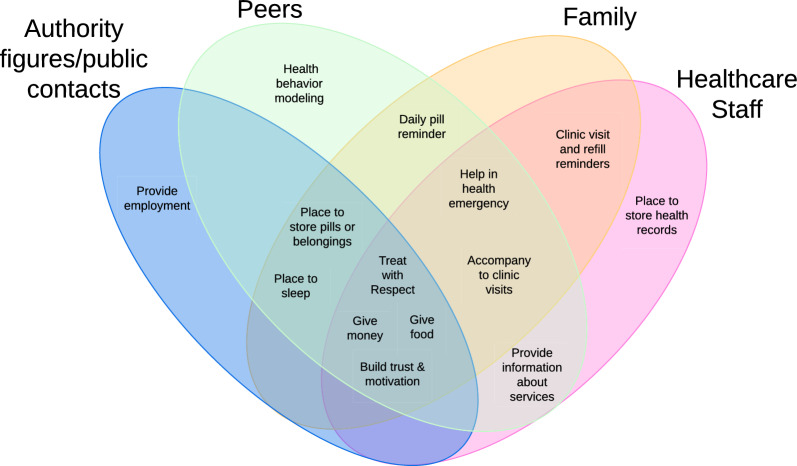
Overlap of health management support from social ties with PWID. *Note*: social ties also contributed challenges to health management which are not shown in figure

Participants often experienced isolation but identified people in their lives who they interacted with. They discussed ways that these social ties both created challenges for their health and wellbeing, as well as ways that they offer support. Types of social connections included healthcare staff who participants saw when they attended clinic or study visits; peers such as friends or injecting partners; family members; and authority figures/public contacts including employers, police staff, shopkeepers, or street vendors. The support provided by these social ties overlapped in many ways across the kinds of social support they offered (see Fig. 1). Figure 1 displays the specific types of support participants discussed in interviews, organized to show the overlap across categories of social connections (represented with ovals) who provided each type of support. A type of support which falls in multiple ovals demonstrates that multiple categories of social connection provided that support to participants. Based on the examples of social support which participants discussed in interviews, there were certain kinds of support which all four types of social ties provided. These included providing money, providing food, treating participants with respect, and building trust and motivation.

Avenues of social connection also created challenges for participants. In interviews, participants discussed various negative experiences with individuals in their lives. Challenges included experiencing ambivalence, misinformation, disrespect, or violence in social interactions. For each type of social support we focus on in this study (i.e., interpersonal, informational, instrumental, and symbiotic), we present ways that social connections supported participants as well as ways they presented challenges.

### Interpersonal support

Participants talked about interpersonal connections they experienced across types of supporters. These experiences of connection ranged from being treated with respect, receiving motivational messaging for health behaviours, to feeling trust in those who offered social support. Motivational messaging from supporters helped participants carry out health-promoting behaviors, such as eating and taking medicine. These messages came from many types of supporters, including peers. One participant shared a message he received from a peer also living with HIV, “*Keep it up, you are doing a good thing by taking medicines*” (Participant 22, age 38). Another shared, “*[Friends] tell me that ‘Your weight has increased and you have started feeling hungry, keep eating like this. If you don’t eat, you will be in trouble’ and encouraged me a lot*” (Participant 4, age 35).

Some participants with connections with family members received encouragement from family to take medicine regularly and attend healthcare visits for their own wellbeing. A participant recalled his brother saying, “*Brother, I got stuck with this kind of disease, and this is how I am taking the medicine now. Do this. See, life is everything*” (Participant 18, age 28). Another participant shared that his mother explained, “‘*[Doctors] don’t give anything without any reason. You are a fool that you are not eating this expensive medicine. They are calling you and giving you medicines to treat you, you should have it.’ Then I started to take these medicines*” (Participant 19, age 27). One participant’s grandmother ensured that he took medicine daily and at a fixed time, “*Even if I have to go out, my grandmother says, ‘You won’t get late, will you? Take the medicine along’”* (Participant 16, age 30).

Some participants shared that they received emotional support from family members and close friends after disclosing their HIV status and confiding in them about injecting drugs. One participant shared that his brother supported him after he revealed his HIV status, “*He is also the one who loves me the most at home. He has also told everyone at home clearly, let him enjoy life*” (Participant 16, age 30). One participant shared how he has built trust with a shopkeeper who offers him multiple sources of social support, “*On disclosure of my positive status he said that there is no problem. You are HIV positive, you should take the medicines daily, and there won’t be any issue later. I can trust him. He is a good person. He tells me to eat and drink”* (Participant 14, age 30).

Participants appreciated how staff at the trial study site were polite and respectful, interacting without discrimination or judgement. “*Everything boils down to the person. They don’t talk to anyone rudely… And even if they have, explaining it to them with love is the biggest thing…then one even follows what they are saying”* (Participant 16, age 30). Treating clients with love and respect, and “*treating you like their own*” (Participant 20, age 30), made participants want to come back to the study site and have future interactions with staff. Participants described these interactions as notably different than how they are often treated by people in authority. Participants also received encouragement from health staff about taking medicines daily. “*Their scolding me was perfectly right. It was good for me. If they hadn’t scolded me, I wouldn’t have started my medication”* (Participant 2, age 30). Clients shared that trial study staff encouraged them to think about health and goals in the long-term. *“They explain to everyone that if you follow the instructions, you will remain in your place, life is in your hands now, let it be, it is your wish”* (Participant 11, age 21). Participants highlighted the impact of receiving positive messages about the future, *“I liked the fact that [the outreach worker] told me about HIV medicine. She said to me, this is the kind of thing HIV is… My life could get better”* (Participant 18, age 28).

### Interpersonal challenges

Participants shared how their interactions with peers could lack substantive connection or turn hostile. “*Friends are there. But they are just for the namesake. Only friends are with you if you have money…But once the money is over they will go away. When you don’t give them, they will abuse*” (Participant 21, 30). One participant questioned the value of friendship in health management, “*Who are such friends, who will be with me and not do their work? They can’t be with me 24/7, reminding me to take the tablet*” (Participant 5, age 22). Participants shared various instances of peers stealing belongings, which limited interpersonal connection and trust. *“I only talk to a few people, they are all thieves. They took my money once”* (Participant 3, age 22).

Instead of interpersonal support, some participants received ambivalence from family. One person shared that his family members do not take interest in his life, “*They just see me taking my medicines sometimes. Otherwise, they are not aware of what medicines I have and when. They are busy on their cell phone all the time…Nobody pays attention to me. I have to do it myself”* (Participant 2, age 30). For others, interactions with family members were distinctly negative, including insults and violence. For example, “*I used to tell [my mother] that if I didn’t take smack I will die. She would say I can go die*” (Participant 17, age 33). The same participant shared a particularly aggressive relationship with his brother, “*My brothers get violent with me. He beats me and if someone asks what happened, then he would say that I got intoxicated and fell down. I can’t say anything in front of him or I am a drug addict and jobless*” (Participant 17, age 33). Disrespect from family was sometimes received in relation to participants’ HIV status. A participant mentioned how he had to keep his belongings separate, “*I can’t touch anything that belongs to [my family]. If I touch a utensil at home, my sister will immediately ask me why did I touch it and starts cleaning it*” (Participant 2, age 30).

### Informational support

Sharing information was a valuable way that social connections supported participants. Peers of participants who were also living with HIV extended support by providing information about how to access healthcare, such as where and when clinics were located and what services they offer. Some peers went beyond verbally sharing information to modelling health behaviors and accompanying participants to services, *“I have a friend, he had taken me with him to get myself tested for HIV. He would tell me get tested and you are surely going to find out that you have this disease. I went with him. I got myself tested and found out that I had this disease for real”* (Participant 9, age 30). One participant discussed how peers share information about taking medicine and preventing transmission, “*As my friend also has HIV, so he also keeps sharing things [about taking medicine]. I also share [information] with those who don’t have HIV. So they don’t use one another’s [syringe]”* (Participant 1, age 22).

Healthcare staff from the study trial site also provided informational support to participants about accessing health services. One participant said, “*[The outreach worker] came with me…I don’t have knowledge about these things. I had never done [HIV testing] before. At least one person should be there with me”* (Participant 19, age 27). Participants were accustomed to healthcare environments which feel unwelcoming and confusing, so they appreciated receiving accessible information about how and where to be seen by a provider. In many cases, study staff accompanied participants to locations for healthcare visits which helped in making sure they knew where to go and how to navigate a clinic. “*The [hospital] guards would not even let [people who live on the street] in…They will dispel them. If someone takes them along, then they cannot dispel them. Then the person tells them that he is with him, he is from the NGO”* (Participant 9, age 30). Healthcare staff from the study also provided help to understand information on medical records and if desired by participants, stored their records at the clinic.

### Informational challenges

Peers also had a negative influence on health behaviors with the information they shared. For many participants, peers created a channel to access drugs and created opportunities to inject. “*There were some friends of mine who used to take the needles. They used to ask me to try it as well, I kept denying. But I relented and tried it for the first time and got stuck in it ever since*” (Participant 19, age 27). While participants learned about health-promoting behaviors from peers, they also heard misconceptions about medications and disease. The same participant recalled, *“Some boys used to taunt me saying ‘they are just misguiding you with the medicines…you don’t have any HIV, it is all a lie.’ So I got distracted and didn’t take any medication initially”* (Participant 19, age 27).

### Instrumental support

Supporters also extended help by providing tangible commodities including money, food, medicine, or a place to sleep for participants. One participant discussed how his mother always reminded him to take medicines after dinner and would give him money to buy milk since he drank milk before taking the medicines (Participant 19, age 27). Peers of participants sometimes extended help by arranging to retrieve food or medicines. One participant talked about his only friend, “*He helps me if I tell him to bring medicine for me from the store, he will get it. If I tell him I can’t move and I need food, he will get me some food as well”* (Participant 7, age 21). In another instance, a participant shared that if he had only a few days’ medicine left, he would ask others he knows who take the medicine, “*If you have any extra pill, give me a pill or two as my medicine supply is going to end soon”* (Participant 1, age 22). Peers also extended help to each other by providing space to sleep. “*He told me that he doesn’t have a place to sleep. I told him that if he wants, he can sleep with me. We can sleep together”* (Participant 14, age 30).

### Instrumental challenges

While participants generally discussed positive interactions with staff working for the study trial who helped them access commodities, they shared experiences with healthcare staff at other facilities which were sometimes negative. At public hospitals, participants shared how they often had trouble accessing services and faced discrimination, “*We are often told to get lost from here. The guard at the main gate of the hospital doesn’t let PWID enter*” (Participant 9, age 30). If they did receive care at public hospitals, participants shared how staff may not treat them well, “*There they don’t talk to you properly…what they think in their minds, that he is an addict. What can we say?”* (Participant 16, age 30). This made public health services challenging to avail, including retrieving medicine, due to the way participants were perceived by staff.

### Symbiotic support

Multiple participants discussed a mutually beneficial type of relationship among authority figures/public contacts which supported their wellbeing—there was an understanding that each benefited in small ways. Authority figures/public contacts offering such support included flower shop owner, tea vendors, juice vendors, employers, a friend’s mother, and police. In exchange for a small payment, running errands, or work, these connections offered participants something to eat or drink, a place to rest, or space to keep their belongings and store medicine. One participant explained his interactions with a juice vendor, “*He gives me juice for 10 to 20 rupees per glass. Usually he charges 30 to 40 per glass. He doesn’t refuse me. I leave my stuff at the shop. He has other things to do, like he has to order ice. I bring it for him. That’s why he doesn’t refuse me”* (Participant 11, age 21). Another participant discussed his relationship with a shop owner for whom he collects rags from the streets, “*I gather a decent amount from the lane and I sleep outside the shop. I supply goods to the shop owner and in return, he allows me to keep my belongings…I also keep my medicines at the shop”* (Participant 21, age 30). Participants talked about how useful it was to have a reliable, safe place to store their medicines, as they did not have to worry about it getting stolen or about experiencing judgement from others who may see them carrying their pill bottle. As a result, these symbiotic relationships proved helpful in enhancing their experience of managing HIV.

### Symbiotic challenges

Participants also shared examples of the same social connections who provided mutually beneficial support also creating challenges at other times or in other settings. For example, police sometimes helped participants but often maintained discriminatory attitudes. When acting on their authority, police caused violence or put participants in jail. When reflecting on the time that he had been to jail, one participant shared, “*But I hadn’t done anything. They [police] framed me in a motorbike theft case because I look like a thief”* (Participant 17, age 33). Another participant spoke directly about this dichotomy with police,* “There is a police officer. He beats people very badly. I think he is a very good person. He beats people, but he also sends sick people to the hospital immediately”* (Participant 11, age 21).

## Discussion

Findings from 22 interviews with PWID in Delhi, India revealed a variety of ways that social connections both support and challenge HIV management and wellbeing. Family members offered emotional support, financial support, medicine reminders, and trust. Peers also provided emotional support, as well as information about health behaviors and health services and often accompanied participants to healthcare visits. Authority figures/public contacts including employers, shopkeepers, and vendors provided a safe place to sleep or store belongings, which proved crucial to consistently store and take pills. Staff at the parent study site provided respectful encouragement to manage health and provided support to remind and accompany participants to clinic visits. Despite these positive interactions, it is important to recognize that social connections also added barriers to health, such as enabling injecting drug use, stealing medicines, physically attacking participants, and disrespecting participants.

In considering the social ecological model, these data speak to how influential the interpersonal level can be in influencing health behaviors and access to services among PWID. Interviews demonstrated that individual-level factors among PWID—such as internal perception, motivations, and resources—were impacted in various ways, sometimes considerably, by others in their life. However, organizational and structural factors (such as the location of health clinics and societal stigma associated with PWID) still shaped the contexts in which PWID navigated their lives and behaviors. This is in line with the need for public health responses to maintain a multi-level perspective, taking into consideration the interplay between levels across the social ecological model [34].

In literature, types of social support include interpersonal connection, informational support, and instrumental support [15]. When sharing ways that social connections promoted health behaviors and wellbeing, participants described experiences which fit these domains. Helpful interpersonal connections included being treated with respect, messages which prompted health behaviors such as taking pills, and building trust. Social connections provided information about where and how to access services and provided access to commodities, such as money, food, and medicine. Participants also discussed an additional domain of support—symbiotic support. In these mutually beneficial relationships, social connections provided support to enhance health management in exchange for favours or work offered by PWID. In interviews, PWID discussed how symbiotic relationships enabled them to store their medicines in a safe location and maintain medicine adherence. Future work can explore this novel type of social support to better understand ways to leverage these symbiotic relationships to enhance health outcomes for PWID.

Accessing potential benefits of social support for disease management may depend on disclosing to others that one is living with a disease. Participants in our study shared encouraging instances of receiving support and care from people who they disclosed their HIV status to, yet this may not be easy or smooth in every case. Given high levels of stigma surrounding HIV, studies have shown that people living with HIV often worry about others knowing their status and choose not to disclose it to protect oneself from judgement [13, 41, 42]. A 2016 study in Vietnam among PWID explored how social support is related to disclosure of disease status, reporting that PWID’s willingness to disclose their HIV status may be dependent on their ability to share information without risk of additional stigma [43]. Despite these barriers, studies report how disease status disclosure can bring benefits of increased social support to help manage the disease such as transportation to health facilities or assistance with paperwork [9, 43], adding to the benefits discussed by participants in our study. This highlights the importance of stigma-reduction efforts at community, institution, and society levels to create contexts which facilitate disease status disclosure. Until HIV stigma is greatly reduced, the benefits of social support for individuals’ health management will be limited [9].

Evidence from our study as well as past literature support the opportunity for public health programs to explore avenues of social support for PWID to promote their health management and wellbeing as a harm reduction strategy [44]. Instead of providing unwelcoming or even dehumanizing experiences for PWID, public health service staff have an opportunity to offer care in an environment which brings a social support system and builds trust through respectful, nonjudgemental support [45, 46]. Healthcare staff offering services for PWID living with HIV can work to inquire about existing or potential social support avenues from people whose clients interact with in their lives. Conversations can explore a client’s comfort with disease status disclosure and offer examples of ways that different types of social ties may be able to support them in managing their disease and promote wellbeing. However, it is critical for public health programs to integrate the possibility of harm from social connections. Participants in our study shared a few of many possible ways that social ties can create barriers to wellbeing rather than opportunities of support. This adds to literature documenting how negative or stigmatizing interactions limit service access for PWID in India [35]. Public health interventions promoting social support to facilitate health behaviors must embrace this complexity to fully support PWID clients. Staff should pursue conversations about who in clients’ lives are supportive and who may be causing harm, and what strategies are feasible to overcome them. Based on what participants in our study said that they appreciate, repeated interactions with the same staff members and trust built through respectful language can improve efforts of public health programs.

This study is not without limitations. It is important to consider findings in the context of who were included in interviews. All our participants were men, and although the majority of PWID in India are men, women and gender diverse PWID are not represented in our findings. Further, we only interviewed PWID who were already recruited through the parent trial and who had completed at least one follow-up visit. Their experiences may be different than other PWID in Delhi, especially those who have not participated in a trial or are not engaged with any healthcare programs, as participants in trials tend to be more motivated to achieve positive health outcomes. While this paper only presents data on PWID enrolled in the trial, three of the 22 participants interviewed were not initiated on ART at the time of the interview.

## Conclusions

This study adds to a growing body of knowledge about the role of social support in health and wellbeing among PWID. Various types of connections may have an important role to play in providing a supportive environment for PWID to lead healthy and meaningful lives. Especially for PWID living with HIV, social supporters can promote disease management and engagement in ongoing care through interpersonal, instrumental, informational, and symbiotic support. These data support the opportunity for public health interventions and programs to explore avenues of social support for PWID to promote their health—for HIV or other disease management, as well as overall wellbeing. Public health service staff have an opportunity to offer care in an environment which brings a social support system and builds trust through respectful, nonjudgemental support. However, with the potential opportunities of support, social connections may also introduce harm or challenges in the lives of PWID. This duality is critical for public health programs to recognize when exploring avenues of social support as a harm reduction strategy among PWID.

## Data Availability

No datasets were generated or analysed during the current study.

## Abbreviations

ART: Antiretroviral therapy
OST: Opioid substitution therapy
PWH: People with HIV
PWID: People who inject drugs
